# Findings from a web content analysis of resources targeting sporting coaches aimed at educating or upskilling on eating disorders and disordered eating in athletes

**DOI:** 10.1186/s40337-021-00512-7

**Published:** 2021-12-11

**Authors:** Rebecca L. Haslam, Erin D. Clarke, Scarlett Gray, Rachel Gearon, Kirrilly Pursey

**Affiliations:** 1 Priority Research Centre for Physical Activity and Nutrition, University of Newcastle, Callaghan, NSW Australia; 2 School of Health Sciences, College of Health, Medicine and Wellbeing, University of Newcastle, Callaghan, NSW Australia

**Keywords:** Eating disorder, Disordered eating, Athletes, Coaches, Web analysis

## Abstract

**Background:**

Eating disorders (ED) and disordered eating (DE) are highly prevalent in athletes. Coaches can play a role in the prevention of EDs and DE behaviours and are well placed to support athletes with an ED. However, coaches feel under-qualified and lack time and resources for supporting athletes and it is unclear the quality of training and resources available to upskill coaches in this space. Therefore, a web-based content analysis was undertaken to determine the type and source of online education resources currently available to coaches to help identify, prevent, manage and refer on for ED/DE behaviours.

**Methods:**

Three major search engines were searched using a combination of the following terms: (1) DE or ED resource and (2) coaches or sport. Included websites were specific for DE/EDs in athletes; targeted at coaches or sporting organisations; written in the English language; and published by a reputable site.

**Results:**

Twenty four out of 600 websites met inclusion criteria. The main reasons for exclusion were irreputable sites and websites not targeting coaches. The majority of included webpages were from professional bodies (n = 17) and targeted coaches (n = 24) and sporting organisations (n = 15), with an average quality rating of 4.2 out of 6. All websites provided educational resources but none provided official training. The most common topics discussed on these websites was ED/DE signs and symptoms (n = 17), and the effects of ED/DE on performance, mental and physical health (n = 11).

**Conclusion:**

Few reputable online resources were identified in the current review. There is a need for more comprehensive education and training resources aimed at coaches and athletic organisations to help prevent, identify, manage and refer on for ED/DE behaviours.

**Supplementary Information:**

The online version contains supplementary material available at 10.1186/s40337-021-00512-7.

## Plain English summary

Athletes are at a high risk of developing eating disorders or disordered eating. Coaches are well placed to support athletes with an eating disorder, however it is unclear the quality of training and resources available to upskill coaches in this area. This web-based content analysis was undertaken to determine the type and source of online education resources currently available to coaches to help identify, prevent, manage and refer on for eating disorders and disordered eating behaviours. Few reputable online resources were identified in the current review. This highlights the need for more comprehensive education and training resources aimed at coaches and organisations to assist in the identification and management of eating disorders and disordered eating.

**Supplementary Information:**

The online version contains supplementary material available at 10.1186/s40337-021-00512-7.

## Background

Eating disorders (EDs) have one of the highest mortality rates of all mental illnesses worldwide [1]. Eating disorders are diagnosed according to the Diagnostic and Statistical Manual of Mental Disorders 5th edition (DSM-5) [2], with criteria generally relating to restriction of energy intake or binge eating, fear of gaining weight or distorted body image, and/or compensatory behaviours such as purging or vomiting, laxative or diuretic use and excessive exercise. Over the past 20 years, the prevalence of EDs has increased globally with a point-prevalence of 7.8% between the period of 2013–2018, over double that of 2000–2006 [3]. In addition to EDs, rates of disordered eating (DE) behaviours that do not meet ED diagnostic thresholds, such as restricted eating, binge eating, and the use of laxatives or diuretics [4, 5], are also increasing, with the prevalence of DE even higher than ED [6–8]. DE predisposes individuals to developing an ED so it is important to aim interventions at both DE and ED.

Athletes across a range of sports, particularly those that are aesthetic e.g. dance, or weight-dependent e.g. combat sports, are at an increased risk for developing ED or DE due to the strong focus on manipulating energy and nutrient intake for the purposes of making weight, manipulating body composition, and improving athletic performance [9]. The prevalence of EDs and DE is higher in both male and female athletes compared to the general population ranging from 6 to 45% in athletes compared to 3–15% of the general population [9]. Variations in DE within the athletic population can be mostly attributed to sex differences, type of sport (e.g. aesthetic, endurance, ball sports or weight dependent) and level of sport (pre-elite or elite) [7, 10–12]. Interestingly, some control populations do report higher prevalence of ED and DE than athletes [13, 14], which may be due to athlete populations under-reporting EDs and DE due to the belief that their restrictive behaviours are the norm [15]. Therefore, appropriate prevention, identification, and management approaches within the sporting environment are essential to identify those at risk.

The behaviours associated with EDs and DE can lead to health complications affecting many body systems including cardiovascular, gastrointestinal, endocrine, and neurological [16, 17]. Such complications may present as rapid weight loss, under-performing, fatigue, poor sleep, loss of menstrual cycle, low mood or irritability, and behavioural signs such as eating in secret [18]. EDs can also affect a person’s ability to perform everyday tasks and maintain positive relationships with others which can adversely impact on their overall quality of life [19, 20]. Health consequences such as electrolyte imbalances, dehydration, mental health issues, increased risks of illness and injury, and reduced training capacity, strength and endurance can all affect athletes specifically [21], as well as severe medical complications including risk of death.

Sporting coaches have frequent contact with athletes and their role within an athlete’s sporting experience is key to their performance and health outcomes [22]. This places coaches in an ideal position to identify and support athletes with DE or EDs [23]. Additionally, coaches can play a preventative role by encouraging positive body image ideals [24, 25] and providing education opportunities for their athletes regarding appropriate nutrition [26]. This is an important role given that body dissatisfaction and weight control behaviours such as dieting are among the strongest contributing risk factors to the development of eating disorders. Evidence has shown that education needs to focus on reducing weight stigma, promoting healthy conversations about weight and eating behaviours, educating about potential health and performance consequences, and how to identify and refer at-risk athletes [27]. However, professional development in EDs and supporting athletes with ED or DE is often not a mandatory part of gaining coaching qualifications [23]. Additionally, coaches often lack time and resources to attend additional training or apply screening procedures [23, 28]. Likewise, sporting organisations have roles in supporting athletes and coaches with respect to promoting a culture that supports healthy nutrition behaviours, positive body image and reducing stigma associated with DE by educating athletes and coaches [15, 29].

There is a growing body of resources available on the internet aimed at educating sporting professionals and organisations on EDs and DE. While there is increasing use of the internet as a source of information, internet searches are currently unable to filter evidence-based nutrition information from pseudoscience [30]. This is concerning given that sporting coaches and organisations may not be equipped with the nutrition literacy to discriminate high quality nutrition and DE evidence. Therefore, it is important to investigate the type and quality of resources available online that target coaches and organisations with the goal of educating on DE and EDs and their associated risk factors.

Therefore, the primary aim of the current study was to undertake a web-based content analysis to determine the type and source of online education resources currently available to coaches to help identify, prevent, manage and refer on for ED and DE behaviours. A secondary aim was to evaluate the overall quality and content of these resources.

## Methods

### Website search

A website search was undertaken in December 2020, using a structured protocol developed by the research team, informed by previous studies [31, 32]. The search included three primary search engines (Google, Yahoo and Bing). These three search engines were used to allow a broad scope of websites and to ensure a complete review of content for those looking for information or support in the area of DE and EDs in athletes. In addition, a comparative analysis of search engines stated that Google, Yahoo and Bing were the most utilised search engines [33]. This study reported that directories (a collection of human reviewed websites arranged by topic) can impact the search results on each engine and when using different search engines, the results are varied in each search. For this reason, it was important that three search engines were used.

The search terms for the website review included two sets of terms used in combination: [1] disordered eating resource or eating disorder resource and (2) coaches or sport. These search terms were informed by pilot searches and chosen as they were in line with what coaches or organisations would likely search.

To be included in the web content analysis, the website resources needed to meet the following criteria: (1) The website needed to be specific for DE and EDs in athletes including information about identification, prevention, management, and referral; (2) targeted at coaches or sporting organisations; and (3) written in the English language. All levels of sport (i.e. recreational, collegiate, and competitive) for individual and team athletes were included. Only resources published by a reputable site were included in the review including sporting organisations, health organisations or an individual provider such as an Accredited Practising Dietitian, Psychologist or Medical practitioner.

Exclusion criteria were: blogs that were not from reputable sources, journal articles, commercial websites, paid advertisement and gym publications, and websites that were not specific to EDs and DE in athletes. Blogs and gym publications were first screened to determine if the information was provided by an individual with a suitable health-related qualification e.g. medical practitioner, dietitian or psychologist, before determining eligibility in the review. Those written by health or fitness organisations were not included if they were not written by an author with a health-related qualification. Journal articles were excluded as the aim of the study was to identify freely available, consumer resources rather than academic-focussed publications.

A pilot search was conducted to determine the number of websites that would be included in the screening for each search. The first 50 results were screened during the pilot test. From this it was determined that only the first 20 websites would be included in the screening process as after the first two pages no websites met the inclusion criteria. This allowed screening to be consistent with other website analysis papers which have noted that it was rare for an individual to search past the first two pages [31]. Google was searched first and then the same search strategy replicated in Yahoo and Bing. A second researcher completed a quality check of the search to ensure no websites were missed by replicating 25% of the search terms on all three search engines.

### Data extraction

Data extraction was performed by two reviewers (SG and RG) using an extraction template developed specifically for the purpose of the current review by the research team. The type of data extracted included: website description, title information, a summary of information, qualification, origin, type of information, resource description, target reader and referral pathways. The data extraction template was piloted using the first five websites and modifications were made to the data extraction template following review by the research team. Data extraction was then completed for all remaining websites and checked by a third reviewer for any discrepancies.

### Critical appraisal

Included websites were critically appraised using the JBI Critical Appraisal Checklist for Text and Opinion Papers for grey literature [34]. This tool includes six quality assessment statements including items relating to the source and references to existing literature. Websites that satisfied greater than 50% of the quality assessment items (i.e. had a score of four or more out of six) were classed as adequate quality and those which scored three or less were deemed inadequate quality.

### Data synthesis

Once the website review was completed and data extracted, data was synthesised descriptively and reported as a narrative review using summary statistics only. This included number and proportion of resources and reporting of narrative data such as type of resource or qualification of author.

## Results

**Fig. 1:**
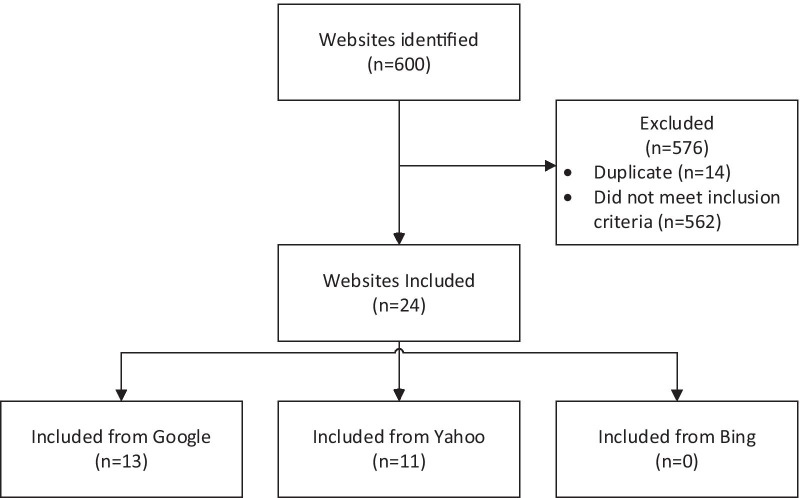
Flow diagram of included websites

The initial search identified 600 websites. Following de-duplication, 24 met the inclusion criteria and were included in the data extraction phase (refer to Fig. 1). Most of the search findings that were excluded were either peer-reviewed journal articles, general blog articles or were not authored by a health professional deemed qualified to educate or upskill on eating disorders. Common websites that were identified as duplicates from the searches on Google, Yahoo and Bing included: National Eating Disorders Association (NEDA), National Eating Disorders Collaboration (NEDC), the Australian Institute of Sport (AIS), Sports Dietitians Australia (SDA), and UK Sport.

The authors of the majority of websites were professional bodies (n = 17), including the NEDC, AIS, SDA, the National Collegiate Athletic Association and the British Association of Sport and Exercise Medicine. Other authors included psychologists (n = 4), doctors (n = 1), exercise physiologists (n = 2), dietitians (n = 1), and counsellors (n = 1) (see Table 1). Only websites from three countries met inclusion criteria, these included the USA (n = 15), Australia (n = 7) and the UK (n = 2). All included websites targeted coaches, while 15 websites targeted organisations as well as coaches (Table 1). No websites were targeted to sporting organisations only.

**Table 1:** Summary of included website characteristics (n = 24)

Website	Year of publication*	Country of origin	Type of website			Qualification of author						Target reader		Type of resource		
			Health Discipline Organisation	Individual Provider	Sporting Organisation	Dietitian	Exercise Physiologist	Psychologist	Counsellor	Doctor	Professional Body	Coaches	Sporting Organisation	Written	Infographic	Video
Website 1 [41]	N/R	USA	X								X	X	X	X		
Website 2 [42]	N/R	USA	X							X		X		X		X
Website 3 [43]	2020	Australia			X						X	X	X	X	X	X
Website 4 [44]	2015	Australia	X								X	X	X	X		
Website 5 [45]	N/R	Australia			X						X	X		X		
Website 6 [46]	N/R	USA			X			X				X		X		
Website 7 [47]	N/R	USA			X			X				X	X	X		
Website 8 [48]	N/R	USA	X								X	X	X	X		
Website 9 [49]	2020	Australia	X								X	X	X	X		
Website 10 [50]	2018	UK	X								X	X		X	X	
Website 11 [51]	N/R	USA	X						X			X		X		
Website 12 [52]	N/R	USA	X								X	X	X	X		
Website 13 [53]	N/R	USA			X						X	X		X		
Website 14 [54]	2017	USA	X			X						X	X	X		
Website 15 [55]	2017	USA	X				X					X	X	X		
Website 16 [56]	N/R	USA			X						X	X	X	X		
Website 17 [57]	N/R	UK			X						X	X	X	X		
Website 18 [58]	2020	USA	X								X	X	X	X		
Website 19 [59]	N/R	USA			X						X	X	X	X		
Website 20 [60]	2014	Australia	X								X	X		X		
Website 21 [61]	2016	Australia			X						X	X		X		
Website 22 [62]	2019	USA		X			X					X		X		
Website 23 [63]	N/R	Australia	X								X	X	X	X		
Website 24 [64]	N/R	USA		X							X	X	X	X		

Website	Year of publication*	Country of origin	Resource characteristics			Referral pathway					Length of resource	
			Educational	Skill Building	Interactive	References	Printable Resources	Additional Resources	Health Professional Referral	None	Brief ^(a)^	Time intensive ^(b)^
Website 1 [41]	N/R	USA	X			X		X				X
Website 2 [42]	N/R	USA	X							X	X	
Website 3 [43]	2020	Australia	X			X	X	X			X	
Website 4 [44]	2015	Australia	X			X		X				X
Website 5 [45]	N/R	Australia	X						X		X	
Website 6 [46]	N/R	USA	X					X			X	
Website 7 [47]	N/R	USA	X							X	X	
Website 8 [48]	N/R	USA	X					X	X		X	
Website 9 [49]	2020	Australia	X			X	X	X	X		X	
Website 10 [50]	2018	UK	X				X	X	X		X	
Website 11 [51]	N/R	USA	X			X			X		X	
Website 12 [52]	N/R	USA	X							X	X	
Website 13 [53]	N/R	USA	X							X	X	
Website 14 [54]	2017	USA	X							X	X	
Website 15 [55]	2017	USA	X							X		X
Website 16 [56]	N/R	USA	X	X	X		X	X	X		X	
Website 17 [57]	N/R	UK	X			X		X	X			X
Website 18 [58]	2020	USA	X			X		X	X		X	
Website 19 [59]	N/R	USA	X			X		X	X		X	
Website 20 [60]	2014	Australia	X					X	X		X	
Website 21 [61]	2016	Australia	X			X		X	X		X	
Website 22 [62]	2019	USA	X			X		X			X	
Website 23 [63]	N/R	Australia	X					X	X		X	
Website 24 [64]	N/R	USA	X			X		X	X			X

(a) Brief—several pages and could be read in one sitting. (b) Time intensive—more intensive resource, which would require more time to read e.g. a manual or website with lots of embedded links to further information

*Year of publication refers to the year that the webpage was last updated or resource developed if this information was available

The quality assurance check identified that twenty of the 24 included websites were scored as adequate, with scores ranging from 3 to 6 (mean 4.2) refer to Additional file 1. The main reasons for lower quality scores was not naming an author(s) (n = 16) and no reference to the existing literature through a reference list (n = 17). Question 6 on the checklist was marked as yes for all, as none of the websites provided any information that was incongruence with the existing literature, therefore no defence of incongruence was needed. Those of higher quality clearly identified the source of the opinion and the source had good standing in the field of eating disorders. Higher quality websites also ensured the interests of the relevant population were the central focus of the opinion, had strong logic in the opinion expressed and made reference to the extant literature.

All websites provided a written resource; however, some websites also provided additional resources (Table 1) including an infographic (n = 2), or a Youtube video (n = 2). The information provided on websites was most commonly presented as a webpage only (n = 14), a downloadable resource e.g. a PDF or infographic (n = 6), a toolkit (n = 3), or a YouTube video (n = 2). All websites (n = 24) provided educational resources; with one also providing an interactive resource and one a skill building resource.

**Fig. 2:**
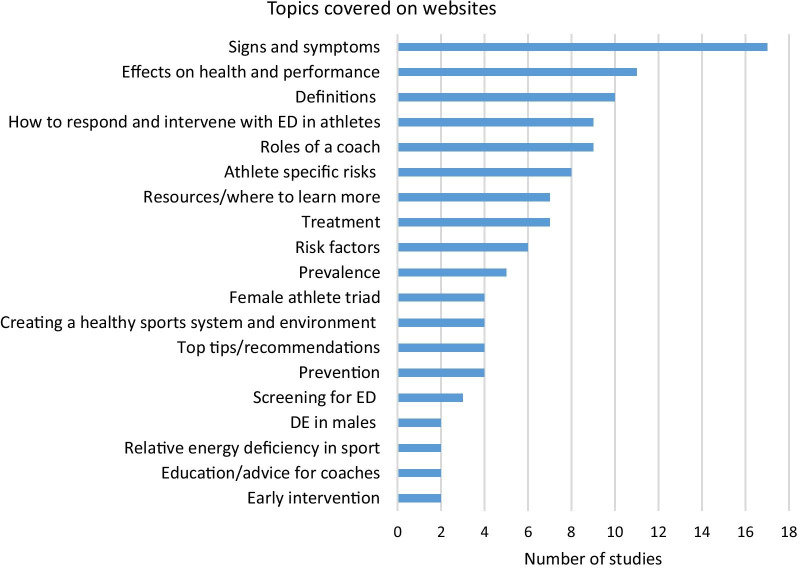
Summary of topics covered on websites related to eating disorders and/or disordered eating in athletes

The content covered across websites was varied (Fig. 2). The most commonly covered topics on ED and/or DE included: signs and symptoms (n = 17); the effects of ED/DE on performance, mental and physical health (n = 11); definitions of ED/DE (n = 10); risk factors (n = 6); athlete-specific related risk factors (n = 8) treatment (n = 7); and where to learn more (n = 7). Some websites specifically focused on information for coaches and sporting communities and included topics such as: the role of the coach (n = 9); how to respond and communicate to athletes about ED (n = 9); education and advice for coaches (n = 2); and how to promote a healthy sport system (n = 4). Only a fifth (n = 5) of the websites provided sex-specific risks; all other websites were targeted at males and females together. Other websites focused on more sport specific conditions such as relative energy deficiency in sport (RED-S) (n = 2), and low energy availability (LEA) (n = 1). Only one website targeted its content toward aesthetic sports specifically, with all other websites targeting athletes in general. A detailed summary of the target reader and content included can be found in Table 2.

**Table 2:** Summary of target reader and content included in website resources

Website	Target reader	Was content sex-specific	Content included
Website 1 [41]	For gyms, school settings, outside athletics groups, dance studios	No	A toolkit for coaches and trainers; 57-page document on prevention, early intervention, treatment as well as education for coaches, definitions of ED, DE, signs and symptoms, roles of a coach
Website 2 [42]	Targeted at coaches who coach, collegiate athletes	No	Information on how to support athletes with eating disorders including role of coaches, signs and symptoms, risk factors, how coaches can educate and be roles models
Website 3 [43]	Targeted at those involved in any kind of sport that is high performance	No	A call to action for those who are involved with sport. Information about ED and DE and how that affects mental and physical health of high-performance athletes. Includes signs and Symptoms, FAQ, creating a healthy sport system, identification and prevention toolkit
Website 4 [44]	Targeted at sports and fitness- sports, clubs	No	Prevention and early identification and response for EDs in sport, how to promote health and wellbeing within sport, how to recognise and respond to EDs, how to work with an athlete with an ED, where to learn more
Website 5 [45]	Targeted at Australian coaches, coaching at any sport level with males or females	No	Information on what are eating disorders, risk factors for eating disorders, related risks, medical and performance complications, treatment approaches, risk reduction strategies
Website 6 [46]	Advice for athletes, parents and coaches- for athletes in aesthetic sports: gymnastics, diving, cheerleading, dance	Includes some female specific information	A 2-page summary of information around ED and DE highlighting the concerns of aesthetic sports. ED risk factors, related pressure, environmental pressures, pressures related to biological / psychological characteristics
Website 7 [47]	Target those who coach student athletes	No	Explores why student athletes are at risk, prevalence, genetics, sociocultural factors, sport related factors, treatment, recommendations to develop a guideline regarding how affected student-athletes are identified, managed and referred for evaluation and treatment by sport personnel
Website 8 [48]	Targeted at athletics directors, coaches and trainers	No	A one-page summary focusing on eating disorders and DE in athletes including ED signs and symptoms specific to an athletic setting, medical consequences of DE and ED in athletes
Website 9 [49]	directed at fitness or sporting professionals	No	A two page downloaded document on what are ED and DE, prevalence and health/ performance consequences, how to identify eating disorders in athletes, what can coaches say and do
Website 10 [50]	Directed at those who teach or coach female athlete’s / dancers	Predominantly female content	Written webpage regarding the complications of ED and DE in athletes. Evolution of female athlete triad to RED-S, LEA, input from coaches/ teachers, what to look for. Includes a downloadable tool for the risk stratification of RED-S
Website 11 [51]	Targeted at those who are concerned in an athlete has bulimia	No	One-page webpage, warning signs, how to help an athlete with bulimia
Website 12 [52]	For coaches, trainers	No	Webpage highlighting why athletes need specialized eating disorder treatment, unique vulnerabilities, unique treatment
Website 13 [53]	Coach and trainers	No	One-page webpage on warning signs of ED inclduing food behaviours, appearance and body image, exercise, thoughts & beliefs, self-harm, social behaviours
Website 14 [54]	Targeted towards more well-educated coaches and trainers	No	Webpage that discusses eating disorders in athletes and the role of coaches in detail. Overview of the issue, how to intervene, warning signs that coaches can look out for including under fuelling/over training, Emphasis on weight, body shape or size, substantial weight loss, disrupted sex hormone cycles and Injuries and other health issues
Website 15 [55]	Targeted at coaches	Some content female specific.	A 23-page presentation for coaches that discusses ED in athletes. types of eating disorders, risk factors of athletes vs non-athletes, signs and symptoms, exercise beyond training, health Concerns, Female Athlete Triad, performance concerns, coach's role
Website 16 [56]	Targeted at those who coach student athlete’s—a high school sport organisation with team sports, track and field, gymnastics and more	No	Webpage that discusses disordered eating in student athletes. Risk factors, complications, treatment, disordered eating scenarios and signs and symptoms. In the scenarios it has 5 questions that describe different the scenario followed by an answer that describes how to approach the situation. There are also resources including sample training day menus and NCAA resources for disordered eating. Incudes reading list & SCOFF
Website 17 [57]	Framework practitioners that work with high performance athletes	No	A 65-page PDF framework including prevalence of ED, characteristic features of eating patterns and clinical syndromes, screening or ED, strategies for practitioners working with high performance athletes, optimum performance weight, making weight, evidence-based treatments, the NHS and accessing therapists, professional competencies and the support team, returning to training and competition during recovery and rehab
Website 18 [58]	Coaches and trainers	No	A 2-page PDF for coaches on what is disordered eating and what is an eating disorder, general signs & symptoms in the sports setting, tips on how to help, referring your athletes for assessment, support and treatment. Provides details of local eating disorder treatment services and discusses that males can be affected too
Website 19 [59]	High school coaches, written by a high school athletics association	No	Webpage aimed at coaches that discusses the prevalence of disordered eating in athletes, signs & symptoms of disordered eating, what sports are at risk for having athletes with DE, prevention, predisposing psychological and social factors, physiological effects, forms of ED (anorexia nervosa etc.) and Female Athlete Triad
Website 20 [60]	Coaches of all levels and sports	No	A 2-page PDF for coaches on what are eating disorders, nutrition and excessive exercise, how to identify eating disorders in sport, what can coaches do, communication
Website 21 [61]	Coaches of recreational athlete’s	No	A webpage that discusses causes of ED in athletes with referencing to NEDC. physical warning signs, psychological warning signs, and behavioural warning signs
Website 22 [62]	Coaches who coach elite athletes	No	Written more like an article including many research papers. Prevalence of eating disorders in athletes, the Female Athlete Triad, Relative Energy Deficiency in Sport (RED-S), disordered eating in males and practical Applications
Website 23 [63]	Sports and fitness professionals	No	How eating disorders can affect sports and fitness professionals, recognise the warning signs, physical warning signs, Behavioural warning signs, psychological warning signs, understanding who is most at risk, screening for eating disorders communication
Website 24 [64]	Educators and coaches of student athlete	No	A Webpage that discusses ED in students and athletes including signs and symptoms specific to school settings, school strategies for assisting students with ED, impact of ED on cognitive abilities and functioning in school, physiologic impacts of bulimia nervosa on athletic performance, free curricular for grades K-12 on ED and healthy body image, info for coaches & trainers, BMI and growth charts for children 2–20 years, confidentiality issues and treatment, common myths about ED, How to start a discussion with someone you think might have bulimia

The majority (n = 19) of websites provided brief information to coaches and organisations on ED and DE characteristics. These resources were short, succinct and primarily used dot points to summarise information. However, five of the websites provided a greater quantity of information through a PDF style manual or PowerPoint presentation. These types of resources would be more time and labour intensive for coaches and organisations to review; however, offered more comprehensive insights.

Almost all of the websites included reference to additional resources or support including linking to ED screening questionnaires, links to organisations which provide assistance/support or names of websites and organisations providing further information, such as referral options for athletes. Included websites also linked to printable resources and over half included a health professional referral for coaches and organisations who were concerned about an athlete, which included dietitians, doctors, counsellors, nurses and psychologists.

## Discussion

The current web-review is the first to investigate the availability of, and content within, online education resources targeted at coaches to help identify, prevent, manage and refer on for ED and DE behaviours. This website content review found that while there are some credible online resources available to coaches, most provide brief information on the risk factors for EDs or DE with few comprehensive resources targeting skill-building or more advanced knowledge of DE. Further, none of the resources were aimed at educating sporting organisations on how to implement education and training within their organisation.

Of the total number of websites identified in the initial search, only a small percentage (4%) were considered suitable for inclusion in this web-content analysis, namely, was suitable for a consumer population and was authored by a reputable individual or organisation. Considering the prevalence of ED and DE in athletes and the role that coaches and organisations can play in the prevention, identification and management of these conditions [23], the lack of evidence-based, consumer friendly, credible resources and websites is a major gap in the area of ED education. Of the resources that were included in this review, many would be suitable resources for sporting organisations to provide their coaches to improve their awareness of the signs and symptoms of ED and DE. Due to the plethora of web-based information where it may be difficult to identify sources of pseudo nutrition, it is recommended that coaches and sporting organisations identify reputable resources and websites by identifying reputable organisations such as SDA, NEDC and NEDA, or qualified professionals such as medical professionals, psychologists or dietitians, who are qualified to provide information on this topic. A strength of the currently available resources is that most of the resources included in this review were rated as adequate quality. However, future resources could improve their quality by clearly identifying the source of the content (author) and making reference to peer-reviewed literature. It is also recommended that these resources be easily accessible with further focus on important information regarding prevention and screening which were covered only in a handful of websites.

While educating coaches on signs and symptoms of ED or DE was a common theme, few resources included information on how to formally screen for EDs and only half included information on how to refer to a healthcare professional, such as a doctor, psychologist or dietitian, if an athlete identified as being at risk of an ED. This is important to include in resources of this kind to assist coaches and organisations to take action for athletes they are concerned about. It is also significant as early identification and treatment is associated with more optimal treatment outcomes [35, 36]. A survey of collegiate coaches in the US identified that coaches most commonly refer to sports medicine personnel within the team, such as athletic trainers or a team physician, rather than refer externally. These team personnel may or may not be trained to manage ED or DE thus influencing the potential management provided [23]. In contrast, a survey of high school coaches in the US identified that 50% or more of coaches would choose to talk with the athlete first or contact the athletes parents prior to referral [37]. In addition, a survey of coaches from the UK identified some disparities in coaches approaches to managing athletes with ED or DE [38], with some involved in the referral of athletes to healthcare professionals while others perceived it was not part of their role. The variable findings in referral and management procedures suggest that coaches and organisations require further support and education regarding the identification of, and referral for, ED and DE [39, 40]. Despite this, resources retrieved in the current review did not provide sufficient information on this topic.

The majority of resources in this web-review provided brief written information on ED and DE. Time has previously been identified as a barrier for coaches to attend further training or undertake further education on ED [28]. Therefore, there are benefits to having brief resources available to educate coaches and sporting organisations on ED and DE. However, for coaches and sporting organisations to obtain a thorough understanding of EDs, including risk factors, physical, mental and performance consequences, and the appropriate screening, communication and referral pathways for EDs, resources need to extend beyond brief written resources. The DE in high performance sport position statement that was released by the AIS and the NEDC proposes that education programs should have multiple targets e.g. coaches, support staff members and athletes, and should be an initial comprehensive education program followed by regular refresher education sessions. Despite this, none of the resources identified in this review provided comprehensive education or training programs, highlighting the urgency for growth in this space if we are to make progress with reducing the prevalence of EDs and DE in sport. Future research including qualitative approaches should assess the needs of coaches and sporting organisations regarding ED education and training to identify the best approaches and information to provide. This should include exploration of the barriers and facilitators to education to appropriately tailor training and resources to the needs of the population.

The higher prevalence of ED and DE in athletes has previously been identified [9], highlighting the need for a cultural shift to occur within sporting organisations as a whole and not just rely on coaches to seek out information themselves. Of the resources identified in this review, no single resource provided sufficient detail about what kind of information is required by organisations and coaches to help with the prevention, identification and management of DE in athletes. Future research should work with organisations and coaches to identify potential barriers and facilitators to change within the system. Additionally, more resources are required to be targeted at the organisational level as it is not only the individual responsibility of coaches, but the whole organisation, to support athletes as well as coaching staff.

There are several strengths to the current review. Firstly, this is a novel study as it is the first of its kind to explore what easily accessible resources are available to coaches and sporting organisations to help them support athletes with ED and DE. This review was modelled from previous web content analysis methodologies and used a systematic approach. It also provides novel insights into the need for more comprehensive education and training to become available for coaches and for sporting organisations to create more supportive environments for the prevention and support of athletes with ED and DE. The limitations of the current review include the restriction to English language only, this may have affected the searches and limited the countries of origin of the websites included. This may mean that the results are not generalisable to countries outside the US, UK and Australia. Only websites deemed reputable were included, this may have resulted in some bias.

## Conclusions

This review identified few reputable online resources for coaches and organisations in the area of eating disorders. Overall, there is a need for more comprehensive education and training resources for coaches and for organisations to help them support athletes with an ED or athletes displaying DE behaviours. Findings from the current review have identified the need for further investigation and discussions with coaches and organisations to determine what information they require, the preferred format of education and training and any barriers and facilitators to implementation. Organisations and health professionals need to be responsive to the needs of coaches to develop appropriate ED and DE resources to further facilitate change in this area.

## Supplementary Information

**Additional file 1:** Critical appraisal of included websites using Joanna Briggs Institute critical appraisal checklist for text and opinion papers.

## Data Availability

All data generated or analysed during this study are included in this published article.

## Abbreviations

ED: Eating disorder
DE: Disordered eating
NEDA: National Eating Disorder Association
NEDC: National Eating Disorder Collaboration
AIS: Australian Institute of Sport
SDA: Sports Dietitians Australia
RED-S: Relative energy deficiency in sport
LEA: Low energy availability

## References

[1] van Hoeken D, Hoek HW (2020). Review of the burden of eating disorders: mortality, disability, costs, quality of life, and family burden. Curr Opin Psychiatry.

[2] American Psychiatric Association. DSM-5 Fact Sheet.2020. https://www.psychiatry.org/psychiatrists/practice/dsm/educational-resources/dsm-5-fact-sheets.

[3] Galmiche M, Déchelotte P, Lambert G, Tavolacci MP (2019). Prevalence of eating disorders over the 2000–2018 period: a systematic literature review. Am J Clin Nutr.

[4] National Eating Disorders Collaboration. Disordered Eating and Dieting https://nedc.com.au/eating-disorders/eating-disorders-explained/disordered-eating-and-dieting/. Accessed 1 April 2021.

[5] Aparicio-Martinez P, Perea-Moreno A-J, Martinez-Jimenez MP, Redel-Macías MD, Pagliari C, Vaquero-Abellan M (2019). Social media, thin-ideal, body dissatisfaction and disordered eating attitudes: an exploratory analysis. Int J Environ Res Public Health.

[6] Vlachakis D, Vlachaki C (2014). Prevalence of disordered eating attitudes in young adults. PeerJ.

[7] Mancine RP, Gusfa DW, Moshref A, Kennedy SF (2020). Prevalence of disordered eating in athletes categorized by emphasis on leanness and activity type—a systematic review. J Eat Disord.

[8] Nagata JM, Garber AK, Tabler JL, Murray SB, Bibbins-Domingo K (2018). Prevalence and correlates of disordered eating behaviors among young adults with overweight or obesity. J Gen Intern Med.

[9] Bratland-Sanda S, Sundgot-Borgen J (2013). Eating disorders in athletes: Overview of prevalence, risk factors and recommendations for prevention and treatment. Eur J Sport Sci.

[10] Sundgot-Borgen J, Torstveit MK (2004). Prevalence of eating disorders in elite athletes is higher than in the general population. Clin J Sport Med.

[11] Martinsen M, Sundgot-Borgen J (2013). Higher prevalence of eating disorders among adolescent elite athletes than controls. Med Sci Sports Exerc.

[12] Karrer Y, Halioua R, Mötteli S, Iff S, Seifritz E, Jäger M (2020). Disordered eating and eating disorders in male elite athletes: a scoping review. BMJ Open Sport Exerc Med.

[13] Patricia Marten D, Carey S (2002). A comparison of female college athletes and nonathletes: eating disorder symptomatology and psychological well-being. J Sport Exerc Psychol.

[14] Wollenberg G, Shriver LH, Gates GE (2015). Comparison of disordered eating symptoms and emotion regulation difficulties between female college athletes and non-athletes. Eat Behav.

[15] Wells KR, Jeacocke NA, Appaneal R, Smith HD, Vlahovich N, Burke LM (2020). The Australian Institute of Sport (AIS) and National Eating Disorders Collaboration (NEDC) position statement on disordered eating in high performance sport. Br J Sports Med.

[16] Peebles R, Sieke EH (2019). Medical Complications of Eating Disorders in Youth. Child Adolesc Psychiatr Clin N Am.

[17] Hetterich L, Mack I, Giel KE, Zipfel S, Stengel A (2019). An update on gastrointestinal disturbances in eating disorders. Mol Cell Endocrinol.

[18] National Eating Disorders Collaboration. Understanding the warning signs https://nedc.com.au/support-and-services-2/supporting-someone/understanding-the-warning-signs/. Accessed 1 Apr 2021.

[19] Malagoli C, Cerro PF, Vecchiato C, Usai MC (2021). Cognitive and emotional regulation in adolescents and young women with eating disorders. Eat Weight Disord.

[20] Ágh T, Kovács G, Supina D, Pawaskar M, Herman BK, Vokó Z (2016). A systematic review of the health-related quality of life and economic burdens of anorexia nervosa, bulimia nervosa, and binge eating disorder. Eat Weight Disord.

[21] Mountjoy M, Sundgot-Borgen J, Burke L, Carter S, Constantini N, Lebrun C (2014). The IOC consensus statement: beyond the Female Athlete Triad-Relative Energy Deficiency in Sport (RED-S). Br J Sports Med.

[22] Davis L, Appleby R, Davis P, Wetherell M, Gustafsson H (2018). The role of coach-athlete relationship quality in team sport athletes’ psychophysiological exhaustion: implications for physical and cognitive performance. J Sports Sci.

[23] Sherman RT, Thompson RA, DeHass D, Wilfert M (2005). NCAA coaches survey: the role of the coach in identifying and managing athletes with disordered eating. Eat Disord.

[24] Sabiston CM, Lucibello KM, Kuzmochka-Wilks D, Koulanova A, Pila E, Sandmeyer-Graves A (2020). What’s a coach to do? Exploring coaches’ perspectives of body image in girls sport. Psychol Sport Exerc.

[25] Smith BE. An exploration of the collegiate coach-athlete relationship and its impact on female athlete attitudes and behaviors toward disordered eating and body image. James Madison University; 2018.

[26] Gumz A, Weigel A, Daubmann A, Wegscheider K, Romer G, Löwe B (2017). Efficacy of a prevention program for eating disorders in schools: a cluster-randomized controlled trial. BMC Psychiatry.

[27] Coelho GM, Gomes AI, Ribeiro BG, Soares EA (2014). Prevention of eating disorders in female athletes. Open Access J Sports Med.

[28] Brown KN, Wengreen HJ, Beals KA (2014). Knowledge of the female athlete triad, and prevalence of triad risk factors among female high school athletes and their coaches. J Pediatr Adolesc Gynecol.

[29] Currie A (2010). Sport and eating disorders—understanding and managing the risks. Asian J Sports Med.

[30] Adamski M, Truby H, Klassen K, Cowan S, Gibson S (2020). Using the internet: nutrition information-seeking behaviours of lay people enrolled in a massive online nutrition course. Nutrients.

[31] Mckenna R, Rollo M, Skinner J (2018). Food addiction support: website content analysis. JMIR Cardiol.

[32] Barnes K, Ball L, Desbrow B (2016). Promotion of nutrition care by Australian fitness businesses: a website analysis. Public Health.

[33] Edosomwan J, Edosomwan T (2010). Comparative analysis of some search engines. S Afr J Sci.

[34] McArthur A, Klugarova J, Yan H, Florescu S (2015). Innovations in the systematic review of text and opinion. Int J Evid Based Healthc.

[35] Le Grange D, Accurso EC, Lock J, Agras S, Bryson SW (2014). Early weight gain predicts outcome in two treatments for adolescent anorexia nervosa. Int J Eat Disord.

[36] Madden S, Miskovic-Wheatley J, Wallis A, Kohn M, Hay P, Touyz S (2015). Early weight gain in family-based treatment predicts greater weight gain and remission at the end of treatment and remission at 12-month follow-up in adolescent anorexia nervosa. Int J Eat Disord.

[37] Kroshus E, Sherman RT, Thompson RA, Sossin K, Austin SB (2014). Gender differences in high school coaches' knowledge, attitudes, and communication about the female athlete triad. Eat Disord.

[38] Plateau CR, Arcelus J, McDermott HJ, Meyer C (2015). Responses of track and field coaches to athletes with eating problems. Scand J Med Sci Sports.

[39] Logue DM, Madigan SM, Melin A, Delahunt E, Heinen M, Donnell S-JM (2020). Low energy availability in athletes 2020: an updated narrative review of prevalence, risk, within-day energy balance, knowledge, and impact on sports performance. Nutrients.

[40] Joy E, Kussman A, Nattiv A (2016). 2016 update on eating disorders in athletes: a comprehensive narrative review with a focus on clinical assessment and management. Br J Sports Med.

[41] National Eating Disorders Association. Coach and athletic trainer toolkit. https://www.nationaleatingdisorders.org/sites/default/files/nedaw18/3.%20CoachandTrainerToolkit%20-%20Copy.pdf. Accessed 22 Jan 2021.

[42] Weltzin T. College coaches and eating disorders: what they need to know. https://www.eatingdisorderhope.com/recovery/self-help-tools-skills-tips/college-coaches-eating-disorders-what-they-need-to-know. Accessed 18 Jan 2021.

[43] Australian Institute of Sport. Disordered eating in high performance sport https://www.ais.gov.au/disorderedeating. Accessed 22 Jan 2021.

[44] National Eating Disorders Collaboration. Eating disorders in sport and fitness: prevention, early identification and response. https://www.nedc.com.au/assets/NEDC-Resources/NEDC-Resource-Sport-and-Fitness. Accessed 18 Jan 2021.

[45] Sports Dietitian Australia. Eating disorders. https://www.sportsdietitians.com.au/sda-blog/. Accessed 19 Jan 2021.

[46] Monsma E. Disordered eating and the controlling aspects of aesthetic sports. https://appliedsportpsych.org/resources/resources-for-parents/disordered-eating-and-the-controlling-aspects-of-aesthetic-sports/. Accessed 22 Jan 2021.

[47] Thompson R. Mind, body and sport: eating disorders. https://www.ncaa.org/sport-science-institute/mind-body-and-sport-eating-disorders. Accessed 18 Jan 2021.

[48] UCSD Eating Disorder Centre for Treatment and Research. Resources for athletic directors, coaches, & trainers. http://eatingdisorders.ucsd.edu/resources/athletics-resources.html. Accessed 18 Jan 2021.

[49] Eating Disorders Victoria. Eating disorder for sport coaches, clubs and teams. 2020. https://www.eatingdisorders.org.au/wp-content/uploads/2020/12/Eating-disorders-for-sport-coaches-clubs-and-teams.pdf. Accessed 19 Jan 2021.

[50] British Association of Sport and Exercise Medicine. Information for coaches/teachers on RED-S.2018. http://health4performance.co.uk/coaches-teachers/. Accessed 20 Jan 2021.

[51] Fielder-Jenks C. Bulimia in athletes. https://www.eatingdisorderhope.com/information/bulimia/bulimia-in-athletes. Accessed 21 Jan 2021.

[52] National Association of Anorexia Nervosa and Associated Disorders. Athletes and eating disorders. https://anad.org/get-informed/athletes-and-eating-disorders/. Accessed 19 Jan 2021.

[53] Towson Sports Medicine. Tips from the athletic training room: eating disorders. https://www.towsonsportsmedicine.com/patient-resources/tips-from-the-athletic-training-room/tips-from-the-athletic-training-room-eating-disorders/. Accessed 20 Jan 2021.

[54] Stranberg M, Quatromoni P. New point of view: athletes & eating disorders. 2017. https://www.waldeneatingdisorders.com/blog/new-point-of-view-athletes-eating-disorders/. Accessed 19 Jan 2021.

[55] Bennett K. Athletes and eating disorders: what every coach needs to know. chrome-extension://gphandlahdpffmccakmbngmbjnjiiahp/https://www.usaswimming.org/docs/default-source/clinics/online-clinic-series/sports-med/4-23-14-athletes-eating-disorders--kate-bennett.pdf?sfvrsn=4.

[56] Association WIA. Disordered eating/relative energy deficiciency in sport. https://www.wiaawi.org/Health/Disordered-Eating-Relative-Energy-Deficiency-in-Sport#43381113-treatment. Accessed 21 Jan 2021.

[57] UK Sport. Eating disorders in sport—a guideline framework for practitioners working with high performance athletes. https://www.uksport.gov.uk/~/media/files/resources/eating_disorders_in_sport.pdf. Accessed 19 Jan 2021.

[58] Interior Health. Eating Disorders & Athletes—a Coach's toolbox. chrome-extension://gphandlahdpffmccakmbngmbjnjiiahp/https://www.interiorhealth.ca/Forms/822123.pdf. Accessed 22 Jan 2021.

[59] Ohio High School Athletic Association. Disordered eating. https://www.ohsaa.org/medicine/healthy-lifestyles/disorders. Accessed 20 Jan 2021.

[60] Eating Disorders Victoria. Eating disorders and sport. chrome-extension://gphandlahdpffmccakmbngmbjnjiiahp/https://www.eatingdisorders.org.au/wp-content/uploads/2019/04/EDV-Eating-disorders-and-sport.pdf. Accessed 21 Jan 2021.

[61] Play by the Rules. Eating disorders and sport. 2016. https://www.playbytherules.net.au/resources/articles/eating-disorders. Accessed 21 Jan 2021.

[62] Virgile A. What you need to know about disordered eating in Sport. 2019. https://adamvirgile.com/2019/02/28/what-you-need-to-know-about-disordered-eating-in-sport-2/. Accessed 18 Jan 2021.

[63] National Eating Disorders Collaboration. Sports & fitness professionals. https://nedc.com.au/eating-disorders/early-intervention/sports-and-fitness-professionals/. Accessed 22 Jan 2021.

[64] Bulimia Guide. Information for educators & coaches. https://bulimiaguide.org/information_for_educators_coaches/. Accessed 22 Jan 2021.

