# Supplementary material for: Findings from a web content analysis of resources targeting sporting coaches aimed at educating or upskilling on eating disorders and disordered eating in athletes
Source: J Eat Disord. 2021 Dec 11;9:159. doi: 10.1186/s40337-021-00512-7 (PMC8665622; doi:10.1186/s40337-021-00512-7)
Supplement: Supplementary file 1 — Additional file 1: Critical appraisal of included websites using Joanna Briggs Institute critical appraisal checklist for text and opinion papers. [file 40337_2021_512_MOESM1_ESM.docx]

**Supplementary file 1: Critical Appraisal of Included Websites using Joanna Briggs Institute critical appraisal checklist for text and opinion papers.**

| **Website** | 1. **Is the source of the opinion clearly identified?** | 1. **Does the source of the opinion have standing in the field of expertise?** | 1. **Are the interests of the relevant population the central focus of the opinion?** | 1. **Is the stated position the result of an analytical process, and is there logic in the opinion expressed?** | 1. **Is there reference to the extant literature?** | 1. **Is any incongruence with the literature/sources logically defended?^#^** | **Score** | **Adequate / Inadequate*** |
| --- | --- | --- | --- | --- | --- | --- | --- | --- |
| Website 1 (41) | Y | Y | Y | Y | Y | Y | 6 | Y |
| Website 2 (42) | Y | Y | Y | Y | N | Y | 5 | Y |
| Website 3 (43) | N | Y | Y | Y | N | Y | 4 | Y |
| Website 4 (44) | N | Y | Y | Y | Y | Y | 5 | Y |
| Website 5 (45) | N | Y | Y | Y | N | Y | 4 | Y |
| Website 6 (46) | Y | Y | Y | N | N | Y | 4 | Y |
| Website 7 (47) | Y | Y | Y | Y | N | Y | 5 | Y |
| Website 8 (48) | N | N | Y | Y | N | Y | 3 | N |
| Website 9 (49) | N | Y | Y | Y | Y | Y | 5 | Y |
| Website 10 (50) | N | Y | Y | Y | N | Y | 4 | Y |
| Website 11 (51) | Y | Y | Y | Y | Y | Y | 6 | Y |
| Website 12 (52) | N | Y | Y | Y | N | Y | 4 | Y |
| Website 13 (53) | N | Y | Y | Y | N | Y | 4 | Y |
| Website 14 (54) | Y | Y | Y | Y | N | Y | 5 | Y |
| Website 15 (55) | Y | Y | Y | Y | N | Y | 5 | Y |
| Website 16 (56) | N | N | Y | Y | N | Y | 3 | N |
| Website 17 (57) | N | N | Y | Y | N | Y | 3 | N |
| Website 18 (58) | N | N | Y | Y | Y | Y | 4 | Y |
| Website 19 (59) | N | N | Y | Y | N | Y | 3 | N |
| Website 20 (60) | N | Y | Y | Y | N | Y | 4 | Y |
| Website 21 (61) | N | N | Y | Y | Y | Y | 4 | Y |
| Website 22 (62) | Y | Y | Y | Y | Y | Y | 6 | Y |
| Website 23 (63) | N | Y | Y | Y | N | Y | 4 | Y |
| Website 24 (64) | N | Y | Y | Y | N | Y | 4 | Y |

* No incongruence was detected therefore no reason to defend. All websites were therefore scored ‘Y; ^#^ Scoring defined as ≥4 yes as adequate and <4 yes as I inadequate

Y = yes, N = no, U = unclear, NA = not applicable
